# High-Dose, but Not Low-Dose, Aspirin Impairs Anticontractile Effect of Ticagrelor following ADP Stimulation in Rat Tail Artery Smooth Muscle Cells

**DOI:** 10.1155/2013/928271

**Published:** 2013-06-06

**Authors:** Grzegorz Grześk, Marek Kozinski, Udaya S. Tantry, Michal Wicinski, Tomasz Fabiszak, Eliano P. Navarese, Elzbieta Grzesk, Young-Hoon Jeong, Paul A. Gurbel, Jacek Kubica

**Affiliations:** ^1^Department of Pharmacology and Therapeutics, Collegium Medicum, Nicolaus Copernicus University, 9 Sklodowskiej-Curie Street, 85-094 Bydgoszcz, Poland; ^2^Department of Cardiology and Internal Medicine, Collegium Medicum, Nicolaus Copernicus University, 9 Sklodowskiej-Curie Street, 85-094 Bydgoszcz, Poland; ^3^Sinai Center for Thrombosis Research, Sinai Hospital of Baltimore, 2401 West Belvedere Avenue, Baltimore, MD 21215, USA; ^4^Department of Pediatric Hematology and Oncology, Collegium Medicum, Nicolaus Copernicus University, 9 Sklodowskiej-Curie Street, 85-094 Bydgoszcz, Poland; ^5^Division of Cardiology, Department of Internal Medicine, Gyeongsang National University Hospital, 79 Gangnam-ro, Jinju, Gyeongsangnam-do 660-702, Republic of Korea

## Abstract

*Objective.* To compare effects of low- versus high-dose aspirin coadministered with ticagrelor on the reactivity of vascular smooth muscle cells (VSMCs). *Methods.* Wistar rats were orally administered ticagrelor (10 mg/kg) and/or aspirin (2 or 10 mg/kg) (*n* = 7 per each of 4 groups) or placebo (*n* = 9) 12 and 2 hours before experiments. Anticontractile effects of ticagrelor were assessed in perfusion solution containing ticagrelor (1 **μ**M/L). Changes in perfusion pressure proportional to the degree of adenosine diphosphate analogue- (2-MeS-ADP-) and phenylephrine-induced constriction of rat tail arteries were evaluated. *Results.* Pretreatment with high- but not low-dose aspirin enhanced the reactivity of VSMCs only in endothelium-lined vessels. Suppression of 2-MeS-ADP-induced VSMC contraction by ticagrelor observed in arteries with and without endothelium was maintained in endothelialized arteries pretreated only with low-dose aspirin. For endothelium-denuded vessels and low-dose aspirin we observed a significant reduction of the maximal effect of ticagrelor with no rightward shift of the concentration-response curve for phenylephrine. With high-dose aspirin pretreatment ticagrelor exerted no anticontractile effect. *Conclusion.* High-dose, but not low-dose, aspirin impairs the anticontractile effect of ticagrelor on ADP-induced VSMC contraction in the rat model. Both the clinical significance and detailed underlying mechanism of our findings require further investigation.

## 1. Introduction

Novel platelet P2Y_12_ receptor inhibitors, prasugrel and ticagrelor, have successfully overcome many pharmacodynamic limitations of clopidogrel and improved outcomes of patients with acute coronary syndromes (ACS) [1–4]. Therefore both ticagrelor and prasugrel have been approved in Europe and in the United States, and their use in the setting of ACS is currently recommended by international guidelines [5, 6].

Ticagrelor is a nonthienopyridine, direct P2Y_12_ blocker that is more potent than clopidogrel and is associated with less interindividual variability in pharmacodynamic effect [7]. Furthermore, important characteristics of ticagrelor, such as rapid onset of action and reversible binding to the P2Y_12_ receptor, may lead to improved outcomes and potentially less bleeding in the setting of urgent surgery.

Substantial reductions in major adverse cardiovascular events and all-cause mortality without significantly increased overall major bleeding complications were observed in the PLATO trial, a landmark phase III study comparing ticagrelor with clopidogrel in a broad spectrum of ACS patients [2]. The unprecedented mortality benefits observed in the PLATO trial, despite only a moderate decrease in the occurrence of myocardial infarction, led to a speculation that ticagrelor therapy was associated with off-target effects [8]. Since P2Y_12_ receptors were identified on vascular smooth muscle cells (VSMCs) [9], we and others have earlier demonstrated in animal and human models that ticagrelor, but not clopidogrel and prasugrel, prevents ADP-induced VSMC contraction [10, 11]. Additionally, other groups have demonstrated that ticagrelor inhibited the uptake of adenosine by human erythrocytes [12] and also induced the release of adenosine triphosphate from human erythrocytes, that is, followed by its degradation to adenosine [13]. The former mechanism was proposed to explain the enhancement of adenosine-induced increase in coronary blood flow observed in a canine model by ticagrelor [12].

A substantial geographic variation in ticagrelor efficacy was observed in the PLATO trial [14]. Based on the results of two independent analyses, it was hypothesized that differences in the maintenance dose of aspirin were responsible for this regional difference. In the subgroup of patients treated with high-dose aspirin (at least 300 mg) in the PLATO trial, particularly in those enrolled in the United States, ticagrelor therapy was paradoxically associated with worse clinical outcomes than clopidogrel therapy [14]. As a consequence of this observation, the US Food and Drug Administration approved ticagrelor with a “Boxed Warning” indicating that aspirin daily maintenance doses of above 100 mg decrease its effectiveness [15].

We compared effects of low-dose versus high-dose aspirin coadministered with ticagrelor on the reactivity of VSMCs in a rat model in this study.

## 2. Materials and Methods

### 2.1. Animals

The experiments were performed on isolated, perfused Wistar rat tail arteries. Animals were housed under a 12 h light/12 h dark cycle and had unlimited access to food and water. Rats (*n* = 37) weighing 250–350 g were pretreated with investigated drugs (*n* = 7 per each of 4 groups) or placebo (*n* = 9), anesthetized by intraperitoneal injection of 120 mg urethane per 1 kg of body mass, stunned, and then sacrificed by cervical dislocation. The study protocol was approved by the Local Ethics Committee, and all experiments were carried out in accordance with the United States NIH guidelines [16].

### 2.2. Drugs and Solutions

The study drugs (ticagrelor and aspirin) or placebo (normal saline) was administered orally 12 and 2 hours before the experiment. Similar to previous studies [10, 11], the doses were ticagrelor (10 mg/kg) and aspirin (2 mg/kg or 10 mg/kg). The doses of ticagrelor and aspirin (2 mg/kg) were equivalent to typical antiplatelet doses in human, whereas the dose of aspirin (10 mg/kg) was comparable to the anti-inflammatory and antipyretic dose used in human studies. Krebs solution contained NaCl (71.8 mmol/L), KCl (4.7 mmol/L), CaCl_2_ (1.7 mmol/L), NaHCO_3_ (28.4 mmol/L), MgSO_4_ (2.4 mmol/L), KH_2_PO_4_ (1.2 mmol/L), and glucose (11.1 mmol/L). All reagents were purchased from Sigma Aldrich Chemical Company (Poznan, Poland) and ticagrelor from AstraZeneca Company.

### 2.3. Study Design and Conduction

**Figure 1:**
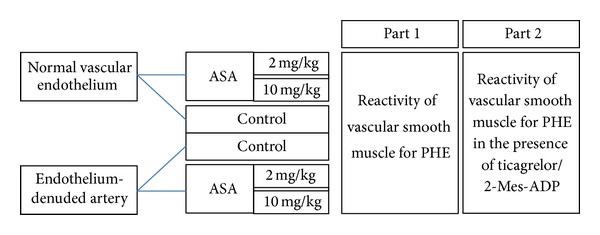
Flow diagram of study design.

After dissection from surrounding tissues, 2.5 to 3.0 cm long segments of rat tail arteries were cannulated and connected to a perfusion device. The distal part was weighted with a 500 mg weight, and the arteries were placed in a 20 mL container filled with oxygenated Krebs solution at 37°C. The perfusion pressure was continuously measured. Perfusion solution flow was gradually increased up to 1 mL/min using a peristaltic pump. Vessel contractions induced with phenylephrine (PHE), a full *α*_1_ adrenergic receptor agonist, in the presence of 2-MeS-ADP (a stable analogue of ADP, 10 *μ*M/L) were measured as an increase in perfusion pressure. The addition of the latter was a consequence of the results of previously published studies indicating that 2-MeS-ADP acts on VSMCs as a partial agonist [10, 11, 17]. The maximal effect after stimulation with 2-MeS-ADP corresponds to 50–60% of contraction evoked by *α*_1_ adrenergic receptors stimulation with PHE or to 60% of contraction induced by KCl (30 mM/L) [10, 11]. Additionally, taking into consideration the reversible binding of the P2Y_12_ receptor by ticagrelor, its effects were assessed both in its presence (1 *μ*M/L) and absence in the perfusion solution. Experiments were performed separately on control arteries and on arteries derived from rats pretreated with low-dose aspirin (2 mg/kg) or high-dose aspirin (10 mg/kg). The experiments were performed separately on arteries with and without vascular endothelium in order to assess the role of the vascular endothelium in regulation of vascular tone in arteries derived from rats pretreated with the investigated drugs (Figure 1). Successful endothelium removal was confirmed by vessel contraction in response to acetylcholine (10^−5^ M/L).

### 2.4. Data Analysis and Statistical Procedures

Concentration-response curves (CRCs) were calculated according to the van Rossum method [18]. The maximal response of tissue (*E*_max⁡_) was calculated as a percent of the maximal response for PHE. Half maximal effective concentration (EC_50_) was estimated using classical pharmacologic methods with pD_2_ the negative logarithm of the EC_50_. We used the number of the CRC and *E*_max⁡_ in all calculations estimating the statistical significance.

Results were presented as means ± standard deviations. The Shapiro-Wilk test was used to determine normal distribution of the investigated variables. Statistical analysis was performed using the Newman-Keuls and ANOVA test for multiple comparisons of means. A two-sided difference was considered significant at *P* < 0.05.

## 3. Results

### 3.1. Effect of High and Low Doses of Aspirin on the Contractility of VSMCs

**Figure 2:**
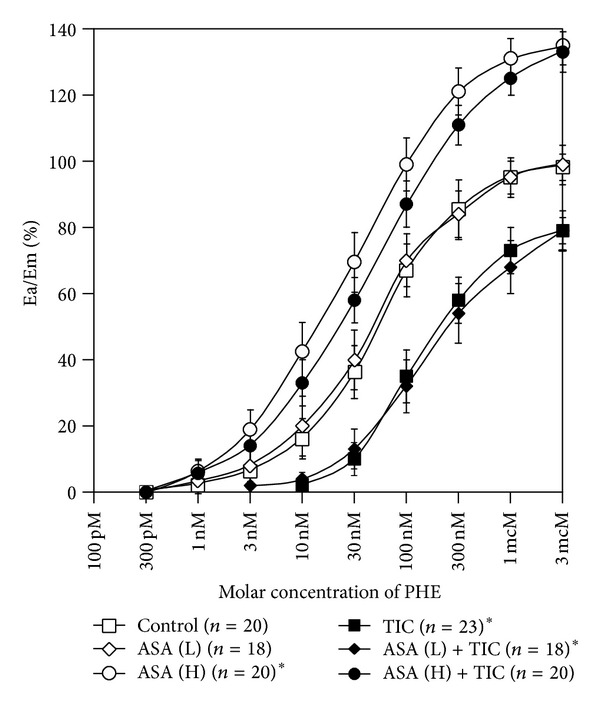
Concentration-response curves in arteries with vascular endothelium obtained for phenylephrine in the absence and presence of low-dose aspirin, high-dose aspirin, and ticagrelor coadministered with 2-MeS-ADP. Points and whiskers display mean values ± standard deviations. A curve for PHE represents a control curve for TIC, ASA (L), and ASA (H) while curves for ASA (L) and ASA (H) constitute control curves for ASA (L) + TIC and ASA (H) + TIC, respectively. ASA (H): high-dose aspirin; ASA (L): low-dose aspirin; Ea/Em: % of maximal response; PHE: phenylephrine; TIC: ticagrelor; 2-MeS-ADP: stable analogue of adenosine diphosphate; *a value of *P* < 0.05 when comparing the control curve for points of effect between 20% and 80% of the maximal response.

**Figure 3:**
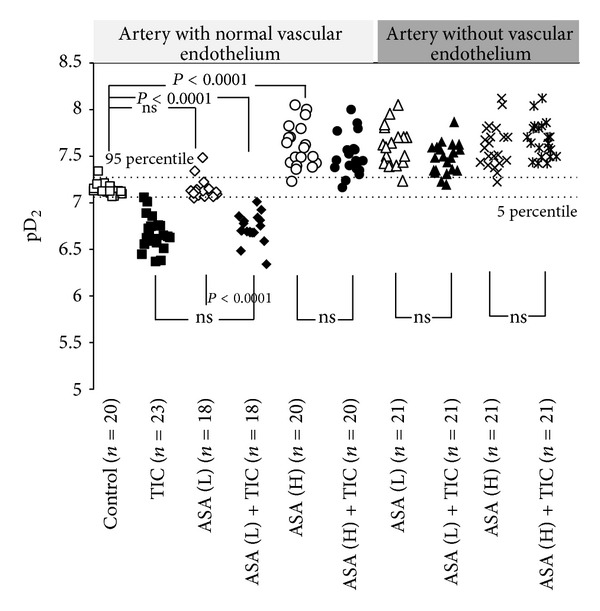
pD_2_ values in arteries with and without vascular endothelium obtained for phenylephrine in the absence and presence of low-dose aspirin, high-dose aspirin, and ticagrelor coadministered with 2-MeS-ADP. Measurements for phenylephrine alone represent controls. ASA (H): high-dose aspirin; ASA (L): low-dose aspirin; Ea/Em: % of maximal response; EC_50_: molar concentration producing 50% of the maximal response; pD2: negative logarithm of EC_50_; TIC: ticagrelor; 2-MeS-ADP: stable analogue of adenosine diphosphate.

CRCs calculated for arteries with endothelium derived from rats pretreated with high-dose aspirin were shifted to the left of control with an increase in the maximal response, whereas CRCs obtained for arteries derived from rats pretreated with low-dose aspirin were comparable to control (Table 1, Figure 2). EC_50_ values in arteries with vascular endothelium calculated for PHE, for rats pretreated with low-dose aspirin and for rats pretreated with high-dose aspirin, were 7.34 (±0.8) × 10^−8^ M/L, 7.33 (±1.4) × 10^−8^ M/L (*P* = ns for comparison with controls—7.34 (±0.8) × 10^−8^ M/L) and 2.62 (±1.4) × 10^−8^ M/L (*P* < 0.0001 for comparison with controls—7.34 (±0.8) × 10^−8^ M/L), respectively. Corresponding pD2 values are presented in Figure 3.

**Figure 4:**
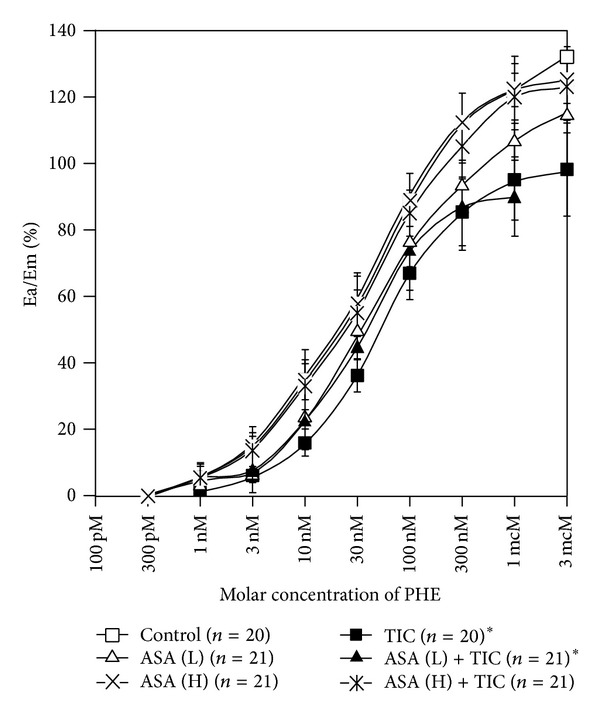
Concentration-response curves in arteries without vascular endothelium obtained for phenylephrine in the absence and presence of low-dose aspirin, high-dose aspirin, and ticagrelor coadministered with 2-MeS-ADP. Points and whiskers display mean values ± standard deviations. A curve for PHE represents a control curve for TIC, ASA (L), and ASA (H) while curves for ASA (L) and ASA (H) constitute control curves for ASA (L) + TIC and ASA (H) + TIC, respectively. ASA (H): high-dose aspirin; ASA (L): low-dose aspirin; Ea/Em: % of maximal response; PHE: phenylephrine; TIC: ticagrelor; 2-MeS-ADP: stable analogue of adenosine diphosphate; *a value of *P* < 0.05 when comparing the control curve for points of effect between 20% and 80% of the maximal response.

When compared with endothelium-preserved vessels, CRCs for PHE in endothelium-denuded arteries were shifted to the left with a significant increase in the maximal response. Neither pretreatment with high nor low dose of aspirin caused any significant shift in comparison with controls obtained for arteries without vascular endothelium (Table 2, Figure 4). EC_50_ values in arteries without vascular endothelium calculated for PHE, for rats pretreated with low-dose aspirin and for rats pretreated with high-dose aspirin, were 1.9 (±1.5) × 10^−8^ M/L, 2.72 (±1.3) × 10^−8^ M/L (*P* = ns for comparison with control arteries without vascular endothelium—1.9 (±1.5) × 10^−8^ M/L) and 2.32 (±0.9) × 10^−8^ M/L (*P* = ns for comparison with control arteries without vascular endothelium—1.9 (±1.5) × 10^−8^ M/L), respectively. Corresponding pD2 values are presented in Figure 3.

### 3.2. Effect of Ticagrelor

The second part of our experiment compared CRCs for PHE (10^−9^–10^−3^ M/L) in the absence and in the presence of 2-MeS-ADP and ticagrelor. In arteries with vascular endothelium, CRCs recorded in the presence of 2-MeS-ADP and ticagrelor showed a rightward shift with a marked reduction in the maximal response, compared to control CRCs (Figure 2). EC_50_ calculated for PHE in the presence of 2-MeS-ADP and ticagrelor (2.35 (±0.9) × 10^−7^ M/L) was significantly higher than EC_50_ for controls (7.34 [±0.8] × 10^−8^ M/L; *P* < 0.0001). The inhibitory effect of ticagrelor was maintained in arteries with endothelial lining pretreated with low-dose, but not high-dose, aspirin (Table 1, Figure 2). EC_50_ values calculated for PHE in the presence of 2-MeS-ADP and ticagrelor in arteries pretreated with low- and high-dose aspirin were 2.03 (±0.8) × 10^−7^ M/L (*P* < 0.0001 for comparison with experiments in the absence of ticagrelor—7.33 (±1.4) × 10^−8^ M/L) and 3.50 (±1.5) × 10^−8^ M/L (*P* = ns for comparison with experiments in the absence of ticagrelor—2.62 (±1.4) × 10^−8^ M/L), respectively. Corresponding pD2 values are presented in Figure 3.

In experiments employing endothelium-denuded vessels, we observed a significant reduction of the maximal effect of ticagrelor without any rightward shift of the concentration-response curve for phenylephrine for low-dose aspirin while any anticontractile effect of ticagrelor was absent for high-dose aspirin (Table 2, Figure 4). EC_50_ values calculated for PHE in the presence of 2-MeS-ADP and ticagrelor in arteries pretreated with low-dose and high-dose aspirin were 3.58 (±1.3) × 10^−8^ M/L (*P* = ns for comparison with experiments in the absence of ticagrelor—2.72 [±1.3] × 10^−8^ M/L) and 2.81 (±1.3) × 10^−8^ M/L (*P* = ns for comparison with experiments in the absence of ticagrelor—2.32 [±0.9] × 10^−8^ M/L), respectively. Corresponding pD2 values are presented in Figure 3.

## 4. Discussion

The main finding of the present study is that high-dose, but not low-dose, aspirin impairs the vasorelaxant effect of ticagrelor on the ADP-induced VSMC contraction in a rat model. This observation provides a new biologically plausible insight into our knowledge regarding the possible interaction between ticagrelor and aspirin doses (North American Paradox). Although the clinical relevance and detailed underlying mechanism of these findings are yet to be determined, coronary vasospasm is generally accepted to frequently accompany atherosclerotic plaque instability and thrombus formation while ADP constitutes an important secondary agonist released from activated platelets. ADP-P2Y_12_ interaction is suggested to be critical for the sustained activation of the glycoprotein GP IIb/IIIa receptor and stable platelet aggregation [4].

In the first part of our study we observed endothelium-dependent increased reactivity of VSMCs after pretreatment with high-dose, but not low-dose, aspirin. The concept linking therapy with nonsteroidal anti-inflammatory drugs (NSAIDs) with the risk of hypertension or worsening of preexisting hypertension has been known for almost 20 years [19, 20]. Blood pressure is determined by peripheral resistance and cardiac output. The former depends on vascular tone while natriuresis intensity constitutes an important factor regulating the latter through changes in blood volume and subsequently in central venous pressure. Despite a clear association between NSAIDs use and rise in blood pressure, the relationship between aspirin and blood pressure is inconclusive [19–27]. A pooled analysis of five cohort studies demonstrated an 18% increase in the risk of hypertension among patients treated with aspirin [21] in contrast to the demonstration of an absence of influence of aspirin dose on blood pressure in two large meta-analyses [19, 20]; moreover, in two other studies, aspirin administered at bedtime lowered blood pressure [22, 23]. Another major confounding factor precluding any definitive conclusion is the range of aspirin doses utilized in the aforementioned studies. Finally, results of numerous trials are in general agreement such that treatment with low-dose aspirin influences neither blood pressure nor the efficacy of antihypertensive treatment [24–27]. Similarly, in our study pretreatment with low-dose aspirin was not associated with any elevation in the vascular tone.

The increased reactivity of VSMCs attributed to the administration of high-dose aspirin in our study may be caused by the inhibition of the prostanoid synthesis. NSAIDs, including aspirin, exert their therapeutic effects by inhibiting cyclooxygenase (COX). Aspirin is believed to exert its cardioprotective properties through irreversible inhibition of the platelet COX-1 isoform and subsequent prevention of the thromboxane A_2_ synthesis while the COX-2 isoform remains a target for classical NSAIDs [28]. However, aspirin, particularly at higher doses, can affect the production of other prostanoids in various tissues [28]. Prostacyclin acting through IP receptors exerts opposing biological effects as compared with thromboxane A_2_, including vasodilatation, suppression of VSMC proliferation, and inhibition of platelet activation. Prostaglandin E_2_ acts through multiple receptors, for example, EP_1_, EP_2_, EP_3_, EP_4_, and IP, and its effects are more variable. In general, prostaglandin E_2_ tends to promote vasodilatation, while at low concentrations it increases platelet reactivity and at high concentrations inhibits platelet reactivity [28]. Inhibition of prostacyclin and prostaglandin E_2_ synthesis by aspirin may result in vasoconstriction and sodium and water retention, thus inducing hypertensive stimuli.

In line with our observations, Aldasoro et al. found that aspirin at high concentrations and nimesulide, a COX-2 isoform selective inhibitor, potentiated the contractile response of gastroepiploic artery to both norepinephrine and vasopressin while low concentrations of aspirin or SC-560, a COX-1 isoform selective inhibitor, did not affect the responses of gastroepiploic artery [29, 30]. Of interest, in other experimental studies, aspirin was simultaneously demonstrated to relax arterial vessels through an increased nitric oxide release from the porcine vascular endothelium by direct acetylation of endothelial nitric oxide synthetase. The latter effect is independent of COX inhibition [31] and through inhibition of PYK2-mediated RhoA/Rho-kinase activation [32].

In the second part of our study we observed that ticagrelor suppresses 2-MeS-ADP-induced VSMC contraction in arteries with and without endothelium. This fact corresponds with a previous report on the identification of P2Y_12_ receptors on VSMCs [9]. The inhibitory effect of ticagrelor was maintained in our study in arteries with endothelial lining pretreated with low-dose, but not high-dose, aspirin. In experiments employing endothelium-denuded vessels, low-dose aspirin treated animals showed a significant reduction of the maximal effect of ticagrelor without any rightward shift of the concentration-response curve for phenylephrine. However, in high-dose aspirin treated animals, ticagrelor evoked no anticontractile effect. Our results suggest that an impairment of the anticontractile effect of ticagrelor by high-dose aspirin and hyperreactivity of VSMCs after pretreatment with high-dose aspirin are strongly influenced by the presence of endothelium. Since the vascular endothelium and VSMCs produce prostacyclin and, to a lesser extent, prostaglandin E_2_ under physiological conditions, we hypothesize that also the interaction between ticagrelor and high-dose aspirin observed in our study may be caused by inhibition of prostanoid synthesis. In line with our hypothesis, FitzGerald et al., using a wide range of aspirin doses, 20–2600 mg, proved that lower doses of aspirin possessed a greater inhibitory effect on thromboxane A_2_ than prostacyclin metabolites, while increasing doses of aspirin suppressed excretion of both thromboxane A_2_ and prostacyclin metabolites in healthy volunteers [33]. Near-complete inhibition of thromboxane A_2_ production by low and high doses of aspirin together with a dose-dependent inhibition of the prostacyclin synthesis was confirmed in other studies of healthy controls or patients with atherothrombotic disease [34, 35]. Additionally, intracoronary infusion of aspirin increased coronary vascular resistance and reduced coronary blood flow [36]. Detailed investigation revealed that intracoronary infusion of aspirin resulted in the attenuation of both pacing-induced coronary hyperaemia and flow-mediated coronary artery dilation [37]. Furthermore, recent studies demonstrated that genomic or pharmacological removal of prostacyclin activated both platelet-dependent and platelet-independent mechanisms inducing atherogenesis, promoting arterial remodelling, and triggering plaque instability and thrombosis [38, 39].

Recent data suggested that aspirin produces little enhancement of platelet inhibition in the presence of strong P2Y_12_ receptor blockade [40]. A recent study showed that ticagrelor is more potent than prasugrel active metabolite in this regard [41]. Ticagrelor may block the P2Y_12_-dependent pathway of platelet aggregation and also sufficiently inhibited platelet aggregation resulting from stimulation with arachidonic acid as suggested by Bhavaraju et al. [42]. Kirkby et al. suggested that in platelets the thromboxane A_2_-dependent pathway is dependent upon the ADP-P2Y_12_ pathway both for the synthesis of thromboxane A_2_ and fundamentally for the irreversible aggregation that follows activation of the TP receptors [41]. It has been speculated that addition of aspirin to a potent P2Y_12_ receptor antagonist may result in side effects secondary to inhibition of COX at nonplatelets sites, thus possibly increasing both bleeding, while providing little additional antiplatelet effects [41]. This concept is in line with the results of the PLATO trial where the occurrence of the primary end point was unrelated to aspirin maintenance dose in the clopidogrel arm [14] suggesting that high-dose aspirin does not offset the effect of clopidogrel, a moderate inhibitor of the P2Y_12_ receptor. The question, whether aspirin provides any additional clinical benefit on top of ticagrelor, should be addressed in future randomized trials.

Our study has several limitations. First, as in all experimental studies, the relevance of the results needs verification in the clinical setting. Despite robust statistical techniques applied by Mahaffey et al., only a randomized study comparing the efficacy of ticagrelor and clopidogrel in relation to aspirin dose could provide a definitive answer. Second, in-depth determination of the mechanism involved in the impairment of the anticontractile effect of ticagrelor by high-dose aspirin warrants further investigation. Third, we did not examine the impact of aspirin dosing on other off-target effects of ticagrelor as they may also contribute to the efficacy of ticagrelor therapy. Finally, the determination of equivalent drug exposures in the rat model versus clinically observed was approximated only.

In conclusion, high-dose, but not low-dose, aspirin impairs the anticontractile effect of ticagrelor on the ADP-induced VSMC contraction in an animal model. Both the clinical significance and detailed underlying mechanism of our findings require further investigation.

## Figures and Tables

**Table 1:** Maximal relative response for phenylephrine in relation to the presence of 2-MeS-ADP, ticagrelor and the dose of aspirin in arteries with vascular endothelium.

	*n* ^1^	*E*_max⁡_ [%]^2^	*P*
Control rats: PHE (10 *μ*M/L)	20	99.0 ± 2.4	
Ticagrelor pretreated rats: PHE (10 *μ*M/L) + ticagrelor (1 *μ*M/L) + 2-MeS-ADP (10 *μ*M/L)	23	79.0 ± 4.7	*P* < 0.0001^a^
Low-dose aspirin pretreated rats: PHE (10 *μ*M/L)	18	99.0 ± 9.8	ns^a^
Ticagrelor and low-dose aspirin pretreated rats: PHE (10 *μ*M/L) + ticagrelor (1 *μ*M/L) + 2-MeS-ADP (10 *μ*M/L)	18	79.0 ± 11.4	*P* < 0.0001^a^ *P* < 0.0001^b^ ns^c^
High-dose aspirin pretreated rats: PHE (10 *μ*M/L)	20	135.0 ± 12.5	*P* < 0.0001^a^
Ticagrelor and high-dose aspirin pretreated rats: PHE (10 *μ*M/L) + ticagrelor (1 *μ*M/L) + 2-MeS-ADP (10 *μ*M/L)	20	133.0 ± 11.7	*P* < 0.0001^a^ *P* < 0.0001^c^ ns^d^

PHE: phenylephrine; 2-MeS-ADP: stable analogue of adenosine diphosphate; ^1^number of concentration-response curves used for calculations; ^2^ *E*_max⁡_: calculated as a percent of maximal response for PHE; ^a^ *P* value calculated versus controls; ^b^ *P* value calculated versus PHE + low-dose aspirin; ^c^ *P* value calculated versus PHE + ticagrelor + 2-MeS-ADP; ^d^ *P* value calculated versus PHE + high-dose aspirin.

**Table 2:** Maximal relative response for phenylephrine in relation to the presence of 2-MeS-ADP, ticagrelor and the dose of aspirin in arteries without vascular endothelium.

	*n* ^1^	*E*_max⁡_ [%]^2^	*P* ^3^
Control rats: PHE (10 *μ*M/L)	20	132.0 ± 14.6	
Ticagrelor pretreated rats: PHE (10 *μ*M/L) + ticagrelor (1 *μ*M/L) + 2-MeS-ADP (10 *μ*M/L)	20	97.0 ± 15.9	*P* < 0.0001^a^
Low-dose aspirin pretreated rats: PHE (10 *μ*M/L)	21	115.0 ± 12.2	ns^a^
Ticagrelor and low-dose aspirin pretreated rats: PHE (10 *μ*M/L) + ticagrelor (1 *μ*M/L) + 2-MeS-ADP (10 *μ*M/L)	21	90.0 ± 13.7	*P* < 0.0001^a^ *P* < 0.0001^b^ ns^c^
High-dose aspirin pretreated rats: PHE (10 *μ*M/L)	21	125.0 ± 12.1	ns^a^
Ticagrelor and high-dose aspirin pretreated rats: PHE (10 *μ*M/L) + ticagrelor (1 *μ*M/L) + 2-MeS-ADP (10 *μ*M/L)	21	123.0 ± 14.2	ns^a^ *P* < 0.0001^c^ ns^d^

PHE: phenylephrine; 2-MeS-ADP: stable analogue of adenosine diphosphate; ^1^number of concentration-response curves used for calculations; ^2^ *E*_max⁡_: calculated as a percent of maximal response for PHE; ^a^ *P* value calculated versus controls; ^b^ *P* value calculated versus PHE + low-dose aspirin; ^c^ *P* value calculated versus PHE + ticagrelor + 2-MeS-ADP; ^d^ *P*-value calculated versus PHE + high-dose aspirin.
